# Zola a Neuropath

**Published:** 1898-10

**Authors:** 


### Zola a Neuropath.

Anthropology—thanks to the labors of Lombroso and other scientific men of the Italian school—occupies a much more prominent position than was at one time accorded it. Max Nordau's startling theory that genius is only a form of degeneration, although not accepted in its entirety, is yet regarded by many as worthy of some consideration. Until recently no genius has offered himself as a subject for investigation. Now, however, Zola has stepped into the breach and has allowed a thorough study of himself to be conducted by a number of French specialists. Whether Zola can be looked upon as a genius is a point upon which probably no two persons will agree, but that he is possessed of abilities far above the average will be conceded by all; and that he has, judging from his writing, many of the mental attributes of degeneration, will also be denied. It appears from the investigations, the results of which are published in a pamphlet by Arthur MacDonald, that physically Zola is somewhat abnormal, but not peculiarly so; that he is neither epileptic nor hysterical, nor is there the slightest sign of mental alienation. Although he has many nervous troubles, the term "degeneracy," does not apply to him wholly. Magnan classes him among those degenerates who, though possessing brilliant faculties, have more or less mental defects. It is true that Zola has orbicular contraction, cardiac spasms, thoracic cramps, false agina pectoris, sensory hyperæthesia, obsessions, and impulsive ideas; his emotivity is defective, and certain of his ideas are morbid; but all this is not sufficient to affect in any appreciable manner his intellectual possesses. His strong and harmonious constitution gives him immunity; his intellect is not contaminated. Toulouse says he has never seen an obsessed or impulsive person who was so well balanced. Yet Zola is a neuropath—that is, a man whose nervous system is painful. Heredity seems to have caused that tendency, and constant intellectual work to have affected the health of his nervous tissues. Now it is a question whether this neuropathical condition is not an excitation that has given rise to the intellectual ability of Zola; whether a diseased nervous system is a necessary cause of great talent or genius is quite another question. Yet pathological facts have been such constant concomitants of great talent and genius that the relation seems to be more than a temporal one, and suggests the idea of cause and effect. However, before this burning question is finally decided, it will be necessary to make a study of several other geniuses.—Editorial *New York Medical Record.*

